# Music-induced emotion as controlled hallucination: an active interoceptive inference account

**DOI:** 10.3389/fpsyg.2026.1759699

**Published:** 2026-02-16

**Authors:** Chen-Gia Tsai

**Affiliations:** 1 Graduate Institute of Musicology, National Taiwan University, Taipei, Taiwan; 2 Graduate Institute of Brain and Mind Sciences, National Taiwan University, Taipei, Taiwan

**Keywords:** active inference, crossmodal correspondence, interoception, music theory, musical emotion, predictive coding

## Abstract

Music is widely recognized as a powerful elicitor of embodied emotion, yet the precise mechanisms by which auditory patterns are translated into specific bodily feelings remain underspecified. Existing models of contagion and entrainment often lack granular mappings between musical features and distinct interoceptive states. This article proposes a novel theoretical framework viewing musical emotion as an instance of active interoceptive inference. I argue that musical structures (e.g., rhythm, dynamics, timbre) function as “pseudo-interoceptive” evidence. Within a hierarchical generative model, the brain integrates these cues with actual physiological signals and extramusical context to infer the somatic state of a “virtual body” implied by the music. Conceptually, this approach extends bottom-up theories by emphasizing top-down predictions. It is posited that the resulting conscious experience is a composite: it blends the listener’s genuine physiological arousal—serving as an energetic substrate—with the simulated affective qualia of the virtual persona. To illustrate this, principled mappings are proposed between musical parameters and internal states, specifically focusing on cardiac and pain-like sensations. Analyses of works by Mozart, Schubert, Berlioz, Beethoven, and Verdi demonstrate how composers manipulate these cues to drive a relatively high level of precision-weighted prediction error, thereby sustaining attention and fostering immersion as the music unfolds. Ultimately, this framework redefines music-induced emotion as a “controlled hallucination” of bodily change, offering new insights into aesthetic empathy and the therapeutic potential of music.

## 1 Introduction

Despite decades of research on music and emotion, a fundamental question persists: how do abstract auditory patterns give rise to specific bodily feelings? Listeners do not simply recognize that music is sad or joyful; they often report felt changes in heartbeat, breathing, and muscular tension. This gap between acoustic structure and visceral experience has motivated a range of embodied and simulation-based theories, yet the mechanisms underlying such mappings remain incompletely specified. A prominent class of theories frames music-evoked emotion in terms of embodied simulation. The Mimetic Hypothesis, for example, proposes that listeners understand music via covert motor and subvocal imitation, subtly engaging the musculature of the limbs and vocal apparatus in response to musical structure (Cox, 2016). Similarly, Juslin’s (2013) model of music and emotion highlights emotional contagion and rhythmic entrainment as key pathways: listeners mirror the emotional valence of voice-like acoustic cues and allow internal physiological rhythms—heart rate, breathing, bodily sway—to align with musical patterns. On this view, music does not simply “represent” emotion in an abstract way; rather, it recruits the same systems that underlie vocal expression, movement, and autonomic adjustment.

Neuroscientific work lends support to this embodied perspective. Molnar-Szakacs and Overy (2006) argued that a frontoparietal mirror network encodes musical structure as intentional motor sequences, while the anterior insula links these simulations to the regulation of the body’s internal milieu, thereby contributing to feeling states. Subsequent neuroimaging studies have implicated both mirror-related regions and the insula in decoding musical emotion and in tracking individual differences in empathy and aesthetic sensitivity (Sachs et al., 2018; Koelsch et al., 2021). Yet, despite this growing body of work, existing models rarely specify how particular musical features map onto specific interoceptive states (e.g., cardiac, respiratory, or pain-like sensations), leaving a gap between fine-grained embodied descriptions of musical emotion and mechanistic accounts of bodily feeling.

Interoception is commonly defined as the sensing of the body’s internal condition (Craig, 2002). Contemporary accounts emphasize that interoceptive feelings are not a simple readout of visceral afferents, but inferred states that integrate cardiovascular, respiratory, nociceptive, somatic, and exteroceptive signals with cognitive and affective context (Ceunen et al., 2016; Carvalho and Damasio, 2021). Within this view, interoception is best understood as an integrated, cross-modal percept of bodily state. At the neural level, interoceptive processing is tightly coupled to homeostasis and supported by a hierarchically organized insula–cingulate–prefrontal network. Posterior insula receives homeostatic and visceral inputs, whereas more anterior insula, together with cingulate and prefrontal regions, generates more abstract representations of bodily state and integrates them with exteroceptive information and higher-order appraisal (Carvalho and Damasio, 2021; Damasio and Damasio, 2024). This abstraction and integration imply that interoceptive experience can be shaped by patterns in the external environment. In particular, auditory events that mimic the temporal and qualitative profile of bodily sensations may be incorporated into the interoceptive construct. Consistent with this idea, exogenous simulations of heartbeats—particularly accelerated rhythms—have been shown to modulate interoceptive processing and increase subjective and autonomic arousal (Kleint et al., 2015; Tanaka et al., 2021; Vicentin et al., 2024), indicating that stylized cardiac signals are sufficient to influence emotional and bodily states. From this standpoint, musical sound becomes a candidate source of “pseudo-interoceptive” evidence—evidence that does not originate in visceral afferents but is nevertheless treated as informative about bodily state. In other words, certain acoustic patterns can function as a proxy for interoceptive signals, biasing interoceptive inference and shaping subjective feeling.

Interestingly, historical music aesthetics already anticipated this kind of link between auditory structure and bodily feeling. In the Baroque Doctrine of Affections, musical figures were explicitly theorized as tools for arousing and sustaining specific passions (Buelow, 1973). These embodied associations between musical parameters and bodily states persisted into the pre-Classical and Classical eras and well into the nineteenth century. C. P. E. Bach’s *Fantasia in A major* (H. 278), written during a gout attack, was characterized by Cramer as materializing “flying pain” through rapid figurations (Head, 2016), while Mozart referred to the aria “O wie ängstlich” from *Die Entführung aus dem Serail* as expressing a “throbbing heart,” explicitly linking its musical figuration to cardiac sensation (Bauman, 1991). Such descriptions suggest that European art music did not only aim to convey generic affect (e.g., joy or sadness), but sometimes sought to represent specific interoceptive qualia. From a psychological perspective, these historical accounts can be read as early, informal hypotheses about pseudo-interoceptive mappings—claims that specific musical figures can simulate particular patterns of bodily sensation.

Building on this convergence between contemporary interoception research and historical descriptions of bodily feeling in music, the present article adopts the framework of *active interoceptive inference* as its core theoretical lens (Seth and Friston, 2016). Within this framework, the brain continuously predicts its internal milieu, minimizes interoceptive prediction error through belief updating and allostatic regulation, and subjective feelings arise as the conscious expression of these homeostasis-oriented inferences about bodily state. Against this background, when listeners encounter music containing pain-like or cardiac-like cues, interoceptive generative models are recruited to infer the physiological–affective states of a musical persona—a “virtual body” implied by sound. On this view, emotion contagion during music listening is understood as a consequence of interoceptive generative models minimizing prediction error.

The aims of this article are threefold. First, it formalizes musical emotion as an instance of active interoceptive inference, integrating embodied approaches to music with predictive-coding accounts of perception and affect. Second, it proposes principled mappings between specific musical parameters (e.g., rhythm, dynamics, timbre, harmony, mode) and virtual bodily states, drawing on work on crossmodal correspondences and homeostatic regulation. Third, it illustrates these mappings through analyses of Western classical repertoire, focusing on cardiac- and pain-like qualia and exploring implications for emotion contagion, aesthetic experience, and music-based therapeutic mechanisms. These aims are pursued within a unified theoretical framework that links musical structure to interoceptive prediction and control.

Extending and refining previous theories, the present framework introduces several specific advances. Relative to appraisal-based and categorical models of musical emotion, and to mimetic accounts that emphasize covert motor or vocal imitation, it foregrounds how music can mimic and organize specific bodily sensations (such as cardiac- and pain-like feelings), rather than merely signaling broad emotion categories. It also reformulates the “action program” account of musical feeling proposed by Habibi and Damasio (2014). In their model, music engages evolutionarily conserved action programs for homeostasis and survival: voice-like and rhythmic cues trigger stereotyped autonomic and motor responses (e.g., changes in heart rate, respiration, and skin conductance), and these bodily changes are then mapped by somatosensory and interoceptive cortices into subjective feeling states. The present framework recasts this predominantly bottom-up view within a hierarchical generative model, assigning a portion of the explanatory work to higher levels of the interoceptive system. Finally, the framework exploits the fact that musical works do not present a fixed emotional state but instead unfold over time, proposing that composers can shape the timing and magnitude of musical changes—and the associated prediction errors—to sustain listeners’ attention and deepen their immersion in the evolving musical narrative. From this perspective, rhythmic entrainment as described by Juslin (2013) can be understood not as literal synchronization between the musical beat and the listener’s heart rate, but as the continuous updating of beliefs about the virtual body in response to musical changes that are treated as pseudo-interoceptive evidence.

## 2 Inferring musical interoception

### 2.1 Interoceptive generative models and musical cues

To make the proposed account of musical interoception more precise, this section adopts the predictive coding framework (Friston, 2010; Friston et al., 2017a), in which the brain is cast as a hierarchical inference system: higher levels generate predictions about sensory input based on internal models (prior beliefs), and mismatches between prediction and input (prediction errors) drive either belief updating or *active inference*—implementing actions to minimize prediction error. In this view, covert motor and interoceptive simulations during music listening are not imitation for its own sake, but a means of generating predictions that help minimize prediction error. In this way, predictive coding provides a computational principle for embodied and mimetic accounts of music perception.

Building on predictive coding accounts, Seth (2013) characterized perception as a form of “controlled hallucination.” Here, “hallucination” is used in a technical, non-clinical sense: it refers to the constructive, model-based nature of perceptual experience, not to pathological percepts that arise without constraint. Fundamentally, the “control” lies in the continuous calibration of these generative predictions by sensory evidence and error-correction mechanisms, which differentiates this notion from mental imagery or free imagination (which can be voluntarily generated and need not be anchored to ongoing sensory input). On this view, the brain uses hierarchical generative models to predict the causes of both external and bodily signals, updating those predictions in light of incoming data. Interoception is a special case of this process in which the predicted causes concern the internal milieu, so that subjective feeling reflects precision-weighted inference about bodily state under sensory constraint.

Two influential extensions of this predictive-processing perspective move beyond perception per se, arguing that the same hierarchical generative modeling can be applied to both interoceptive regulation and social understanding. In the active interoceptive inference account (Seth and Friston, 2016), the brain predicts its internal milieu and reduces interoceptive prediction error via belief updating and allostatic regulation, with feelings construed as the conscious expression of these homeostasis-oriented inferences about bodily state. Extending the same logic to theory of mind and social cognition, Ondobaka et al. (2017) proposed that inferences about others’ intentions and emotions are likewise underpinned by hierarchical interoceptive inference: the generative model used to explain one’s own actions and feelings is redeployed to interpret another agent, such that the observer infers which internal states and action policies would best explain the other’s movements and expressions and attributes those inferred states to that agent.

These considerations have direct implications for music perception. Certain musical cues may be assimilated as pseudo-interoceptive evidence, informing the listener’s inferences about the emotional condition of the music-implied virtual body—a simulated persona comparable to a fictional character, rather than a simple set of physical sound properties. For example, rhythmic pulsations resembling the temporal profile of cardiac acceleration may be interpreted as signals of interoceptive fluctuation, prompting the generative model to infer heightened arousal. Subjectively, this can manifest as changes in felt anxiety or urgency that the listener attributes to the music. By analogy with shared-representation accounts of action and emotion (Molnar-Szakacs and Overy, 2006), the present article proposes that insular circuitry—ordinarily used to monitor one’s own bodily state—can be recruited to infer the states of musical persona that best explain the pseudo-interoceptive musical cues. The conscious expression of such inferences is the music-induced emotion, which seems to possess a hallucination-like quality and is considered to belong to the domain of vicarious emotions (Kawakami et al., 2014) or aesthetic emotions, rather than utilitarian emotions (Zentner et al., 2008). Yet these affective experiences are not disembodied. They are likely grounded in neural mappings of bodily states (Habibi and Damasio, 2014).

Notably, this “hallucination” of music-evoked emotion is controlled: its simulated states are continuously constrained and shaped by musical structure, contextual information, and the listener’s ongoing interoceptive and homeostatic demands, rather than drifting freely as unconstrained fantasy. Consequently, this framework focuses on interoceptive *awareness*, broadly understood as the subjective capacity to notice and interpret somatic states. Extending this construct to the processing of pseudo-interoceptive cues from a virtual body, the article proposes a form of musical interoceptive awareness in which listeners can experience specific somatic qualities as structuring features of the music.

In this light, when exteroceptive sounds drive interoceptive inference, music provides a far richer aesthetic and epistemic medium than isolated heartbeat-like stimuli. In experimental studies, such stimuli are often designed to manipulate affect and typically function as decontextualized, quasi-medical inputs (Kleint et al., 2015; Tanaka et al., 2021; Vicentin et al., 2024). By contrast, cardiac-like cues in Western classical repertoire are embedded within harmonic, melodic, and narrative contexts: they are compelling musical events in their own right and simultaneously provide rich contextual information about who is feeling what, and why. This embeddedness not only ensures that the hallucination remains controlled, but more crucially, it renders the musical persona as a living subject inhabiting a virtual world—an entity whose physiological states undergo meaningful and dynamic fluctuations. This depth of engagement helps to explain why listeners are willing to invest sustained attention and emotional involvement in such pieces.

**Figure 1:**
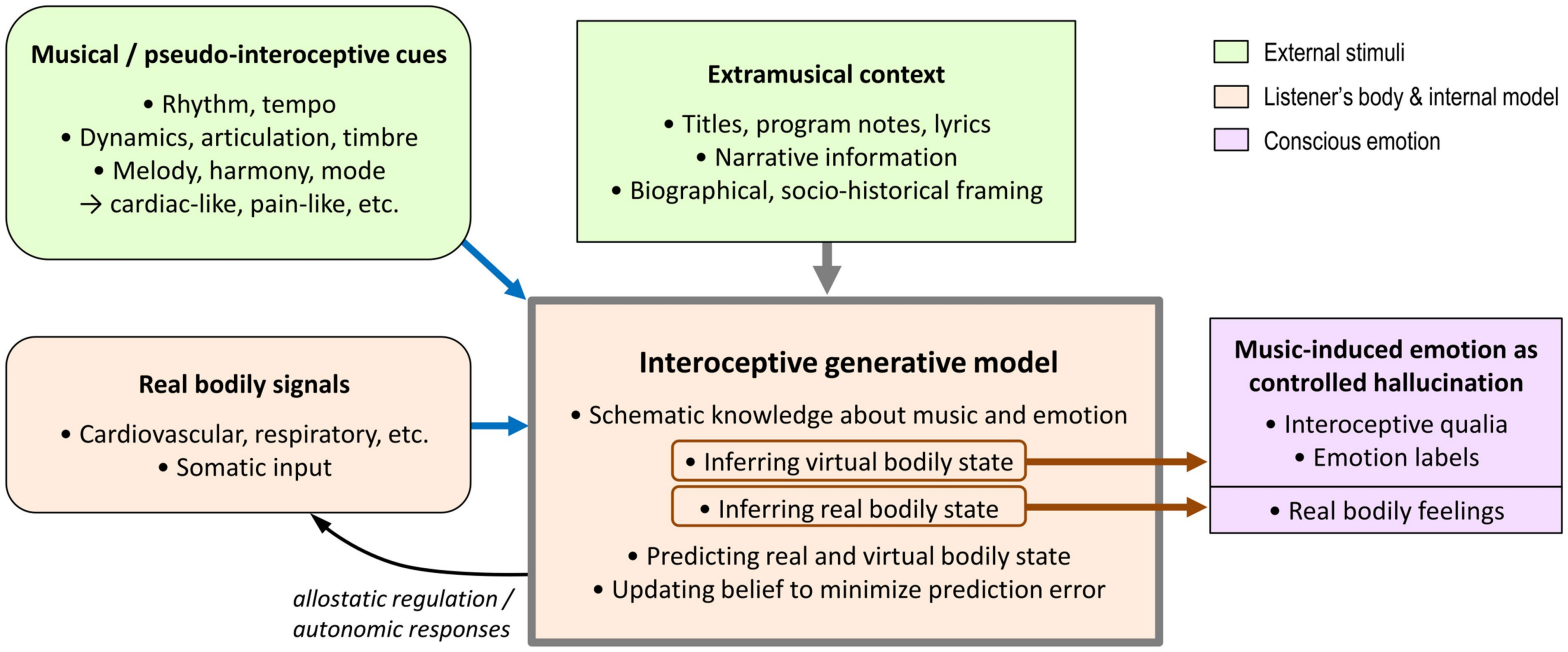
Schematic model of musical emotion as active interoceptive inference. The framework proposes that music-induced emotion arises from a hierarchical generative model (central cream-colored box) that integrates three key sources of information: (1) Musical features (top-left), which function as pseudo-interoceptive cues (e.g., rhythmic pulsations processed as virtual cardiac signals); (2) Real bodily signals (bottom-left), which provide the physiological energetic substrate; and (3) Extramusical context (top-center), which sets high-level priors and constraints. Blue arrows indicate the bottom-up flow of sensory evidence (driving prediction errors), while brown arrows denote the resulting conscious percepts. The generative model minimizes prediction error through belief updating and allostatic regulation (curved black arrow). Crucially, the system infers two parallel states: the state of the virtual body implied by the music and the state of the listener’s real body. The resulting conscious experience (right) is a composite: a “controlled hallucination” of the virtual body’s affective state (interoceptive qualia and emotion labels) anchored by the listener’s genuine physiological feelings.

Figure 1 illustrates the proposed architecture. By integrating pseudo-interoceptive musical cues with afferent bodily signals and extramusical priors, the generative model minimizes prediction error across two parallel tracks: the listener’s real body and the musically implied virtual body. Consequently, the ensuing conscious emotion is a composite experience—a “controlled hallucination” of the virtual persona’s affect that remains grounded in the listener’s actual physiology.

### 2.2 Extramusical information and attribution of emotional meaning

In active interoceptive inference, raw interoceptive signals do not in themselves determine a specific emotion. Instead, they are affectively ambiguous and become meaningful only via context-sensitive inferences about their causes (Seth and Friston, 2016). In line with the Two-Factor Theory of Emotion (Schachter and Singer, 1962), emotion can thus be seen as arising from the interaction between physiological arousal and its cognitive interpretation. This view is compatible with constructionist accounts of emotion, in which core affect is shaped into discrete emotion categories through conceptual and contextual constraints (Barrett, 2017).

In music perception, semantic and extramusical information—titles, program notes, socio-historical framing, and biographical details about the composer—provides a higher-order frame for emotional labeling (Vuoskoski and Eerola, 2013; Kiernan et al., 2021). Such cues constrain how arousal is construed by anchoring ambiguous physiological simulations to specific, consciously experienced emotions (e.g., construing a rapid heartbeat as “romantic longing” rather than “cardiac distress”). In opera and other narrative genres, listeners combine interoceptive cues—including simulated laryngeal sensations associated with the singing voice—with verbal and dramaturgical context to support theory-of-mind inferences about those characters. While the pseudo-interoceptive signals in the music drive emotional Theory of Mind (feeling the persona’s somatic state), extramusical cues support cognitive Theory of Mind (understanding the persona’s situation). Underpinning this entire process, the listener’s genuine physiological arousal serves as the necessary energetic substrate, lending visceral reality to the inferred virtual states.

The present account is developed primarily with Western art music and its often narrative, persona-rich repertoire in mind. The discussion section will briefly consider how far the same principles might generalize to other genres and listening contexts.

### 2.3 Precision weighting, attention, and internal model

A key concept for linking musical structure to attention within predictive coding is precision weighting. Predictive coding models posit that the brain optimizes not only the content of its predictions but also the precision assigned to prediction errors. Precision, typically formalized as the expected inverse variance of a given error signal, determines its impact on belief updating (Feldman and Friston, 2010). High-precision errors exert greater influence, whereas low-precision errors are down-weighted. Functionally, precision modulation can be regarded as a form of gain control on prediction errors and is closely linked to attention. In predictive coding, attention is the process by which the system selectively enhances the precision of certain sensory channels or model components.

Musical organization—particularly the use of variation and contrast—provides a powerful means of shaping precision weighting. In passages dominated by repetition and low informational novelty, the brain learns that its predictions are reliable and that ensuing prediction errors tend to be small. Precision is therefore preferentially assigned to the internal model, while the precision accorded to incoming sensory signals is relatively reduced. Conversely, when a harmonic modulation, textural rupture, or rhythmic change produces a salient prediction error, the prevailing model is momentarily inadequate. To revise its beliefs, the system must transiently increase the precision of sensory prediction errors, thereby amplifying the influence of bottom-up input and reallocating computational resources from entrenched priors to new evidence (Feldman and Friston, 2010). Subjectively, such moments are often experienced as abrupt captures of attention and can heighten perceived aesthetic pleasure. Consistent with this view, Schellenberg et al. (2012) found that listeners generally prefer excerpts exhibiting emotional contrasts over those expressing a single sustained emotion.

The notion of precision-weighted prediction error (pwPE) helps clarify how musical works attract and sustain attention by hitting a “sweet spot” between predictability and surprise. Vuust et al. (2018) argued that rhythmic patterns of intermediate complexity maximize pwPE and are therefore experienced as especially engaging and pleasurable, whereas patterns that are too simple or too complex reduce engagement. Extending this logic, if musical twists occur too frequently, prediction rapidly loses precision, the music becomes effectively unpredictable, and engagement drops.

However, composers do not merely deploy musical twists with caution; rather, they exploit the multidimensionality of music to develop rich techniques for affective contrast. In Western classical music after the Baroque era, composers adeptly redistributed precision across musical dimensions. When precision is lowered in one dimension—for instance, through dissonant chromatic harmony—other dimensions such as motivic repetition typically retain high precision (Tsai, 2024). This strategic counterbalancing ensures that the system maintains a high level of pwPE. By keeping musical surprises salient and the overall structure comprehensible—at times anchored by extramusical context—this mechanism successfully sustains listener engagement.

## 3 Musical features and virtual bodily states

Having outlined the active inference framework, I now turn to the question of why, and in what sense, specific musical features can be mapped onto interoceptive predictions. In perceptual psychology, one relevant line of work concerns *crossmodal correspondences*. Spence (2011) reviewed converging evidence that the human perceptual system exhibits systematic tendencies to associate features across sensory modalities. One class of explanations appeals to *structural correspondences*, which are thought to arise from shared or isomorphic coding principles in the nervous system. Another invokes *statistical correspondences*, which emerge from learned associations based on the frequent co-occurrence of physical properties in the environment. Together, these findings suggest a principled basis for stable mappings between auditory patterns and bodily sensations.

The following sections outline how specific musical features may be mapped onto interoceptive predictions, with a focus on sensations related to cardiac and pain-like states. The same logic may also extend to other kinds of bodily feeling. For clarity, a distinction is drawn between (1) features that primarily specify the physiological state of a virtual body and (2) features that help label and interpret that state as a particular kind of emotion.

### 3.1 Virtual physiological states

*Rhythm/tempo*. Temporal organization maps readily onto somatic rhythms, including cardiac and respiratory rhythms. Fast tempi and dense rhythmic patterns are associated with heightened arousal (van Dyck et al., 2017). Within the present framework, such features may be experienced as analogous to tachycardia or hyperventilation—a descriptive correspondence that invites, but does not entail, a specific mechanistic account. One candidate explanation is that the nervous system encodes temporal information isomorphically, utilizing shared neural codes for external auditory tempo and internal physiological rhythms. Additionally, statistical correspondences play a role: the simultaneous experience of the heart’s tactile beat and its internal sound creates a natural, learned association between these sensory channels.

*Dynamics*. Changes in loudness are typically associated with perceived intensity and suddenness of bodily change. A gradual crescendo tends to evoke sensations akin to mounting palpitations or rising tension, whereas a decrescendo is often experienced as the dissipation of such energy. At the extremes, sforzando attacks may be felt as acute, shock-like intrusions, while an abrupt grand pause can evoke the somatic freezing response—such as the involuntary holding of breath. These descriptive correspondences suggest a systematic relationship between dynamic contour and interoceptive quality, though the underlying mechanism remains to be established. One candidate explanation is that the nervous system encodes changes in intensity in a graded fashion across both auditory and interoceptive domains.

*Articulation*. The manner of sound onset and connection can itself be experienced as a kind of tactile or visceral texture. Behavioral work has shown that staccato melodies are associated with higher perceived tension, energy, happiness, and surprise, whereas legato counterparts are judged as more cohesive and as conveying greater calmness and sadness (Carr et al., 2023). More broadly, research on crossmodal correspondences reviews converging evidence that listeners systematically map auditory features onto tactile- and visual-like dimensions; for instance, Spence (2011) highlighted that high-pitched or abrupt sounds are consistently associated with “sharp” or angular qualities, whereas lower or continuous sounds are linked to “rounded” or smooth forms. Extending this line of work to the interoceptive domain, I propose that staccato and marcato articulations tend to evoke punctate, jump- or stab-like bodily sensations, whereas legato lines are more readily experienced as smooth, fluid, or diffuse. One possible mechanistic explanation is that the nervous system applies similar coding principles to abrupt versus continuous events across auditory and somatosensory domains.

*Timbre*. Spectral shape further refines these qualia. Bright timbres with pronounced high-frequency content are often described as sharp or piercing, whereas darker, low-frequency-rich timbres tend to be heard as dull, heavy, or diffuse. For instance, a piccolo melody, with its high-frequency-rich spectrum, is typically perceived as bright and piercing, while a timpani roll has a darker, booming quality. Extending these observations to the interoceptive domain, I suggest that bright timbres may evoke sharp, localized bodily sensations, whereas darker timbres are more readily experienced as diffuse or heavy—as in the visceral thud of a timpani roll. One candidate explanation for these correspondences draws on statistical regularities in the physical environment: objects with sharp or piercing attributes tend to be composed of hard materials, which emit sounds rich in high frequencies when set into vibration.

### 3.2 Emotional labeling

*Melodic contour*. Pitch motion maps onto implied movement and posture, which in turn invite more active versus passive affective interpretations. Ascending contours tend to suggest effortful reaching, striving, or expansion, and are therefore more often associated with active, approach-like affective interpretations. Descending contours tend to suggest sinking or yielding and are more readily associated with passive or release-like affects. This asymmetry can be understood within the framework of *musical gravity* (Larson and Vanhandel, 2005). I further speculate that it may be partially grounded in laryngeal proprioception: higher pitches typically require greater vocal-fold tension, analogous to a higher level of gravitational potential energy, whereas lower pitches involve reduced tension, analogous to settling into a lower-energy state. This mapping can be partially explained in terms of structural correspondences: the nervous system may use similar coding principles for physiological effort and subjective effort.

*Harmony*. Harmonic configuration can be understood as mapping onto evaluative judgments of the internal environment. Consonance and harmonic resolution signal safety, certainty, and homeostatic recovery—a return toward preferred set points. By contrast, dissonance and harmonic instability signal threat, conflict, or crisis, corresponding to deviations from homeostasis that demand explanation and corrective action. Such mappings can be partially explained in terms of structural correspondences: the nervous system likely encodes the acoustic roughness of dissonance (arising from spectral interference) and the spectral smoothness of consonance isomorphically with somatic states of irritation versus equilibrium. The tension-release pattern in music resonates with *drive reduction* accounts in which deviations from preferred internal states (homeostasis) generate tension and motivate a return toward equilibrium (Hull, 1943).

*Mode*. Within the 18th–19th-century Western classical idiom, mode serves as a fundamental cue for emotional valence, with the major mode signaling positive affect and the minor mode conveying negative affect—a dichotomy supported by robust empirical evidence. This association likely stems from converging factors: the minor mode’s harmonic instability and higher dissonance may evoke uncertainty and tension (Parncutt, 2014), while its lowered scale degrees mirror the prosodic features of sad speech (Curtis and Bharucha, 2010). Crucially, these emotional connotations are not entirely universal but are significantly reinforced by cultural learning and exposure to Western tonal conventions.

## 4 Illustrative analyses of musical works

Building on the proposed mappings, this section applies the framework to five works from the Western art-music repertoire, spanning the Classical to late Romantic periods. By integrating score analysis with extramusical context, I explore how pseudo-interoceptive cues might interact with textual or programmatic constraints to suggest specific somatic meanings. Adopting Spitzer’s (2010) holistic approach, these analyses treat emotions not as static “semantic labels” but as varieties of “emotional behavior” enshrined within musical structure and dynamic trajectories unfolding over time. The examples serve to illustrate the framework’s applicability across two primary interoceptive domains: cardiac sensations and pain-like experiences.

### 4.1 Mozart, *die Zauberflöte* (“dies Bildnis ist bezaubernd schön”): cardiac and respiratory cues in sudden love

In Tamino’s aria “Dies Bildnis ist bezaubernd schön” from Mozart’s *Die Zauberflöte*, the awakening of love is staged as a bodily event, saturated with cardiovascular imagery and layered physiological excitement. Tamino first reports that this portrait of a young woman fills his heart with new agitation, and that this nameless “something” burns in his chest like fire. At precisely the moment he sings the word “Herz” (heart), the accompaniment settles into a clearly pulsating pattern at a moderate tempo, which can be heard as a stylized heartbeat underpinning this newly discovered inner stirring. Once he finally names the feeling as love, the musical pulse grows more insistent, aligning with a subjective sense of accelerated heart rate and mounting arousal.

**Figure 2:**
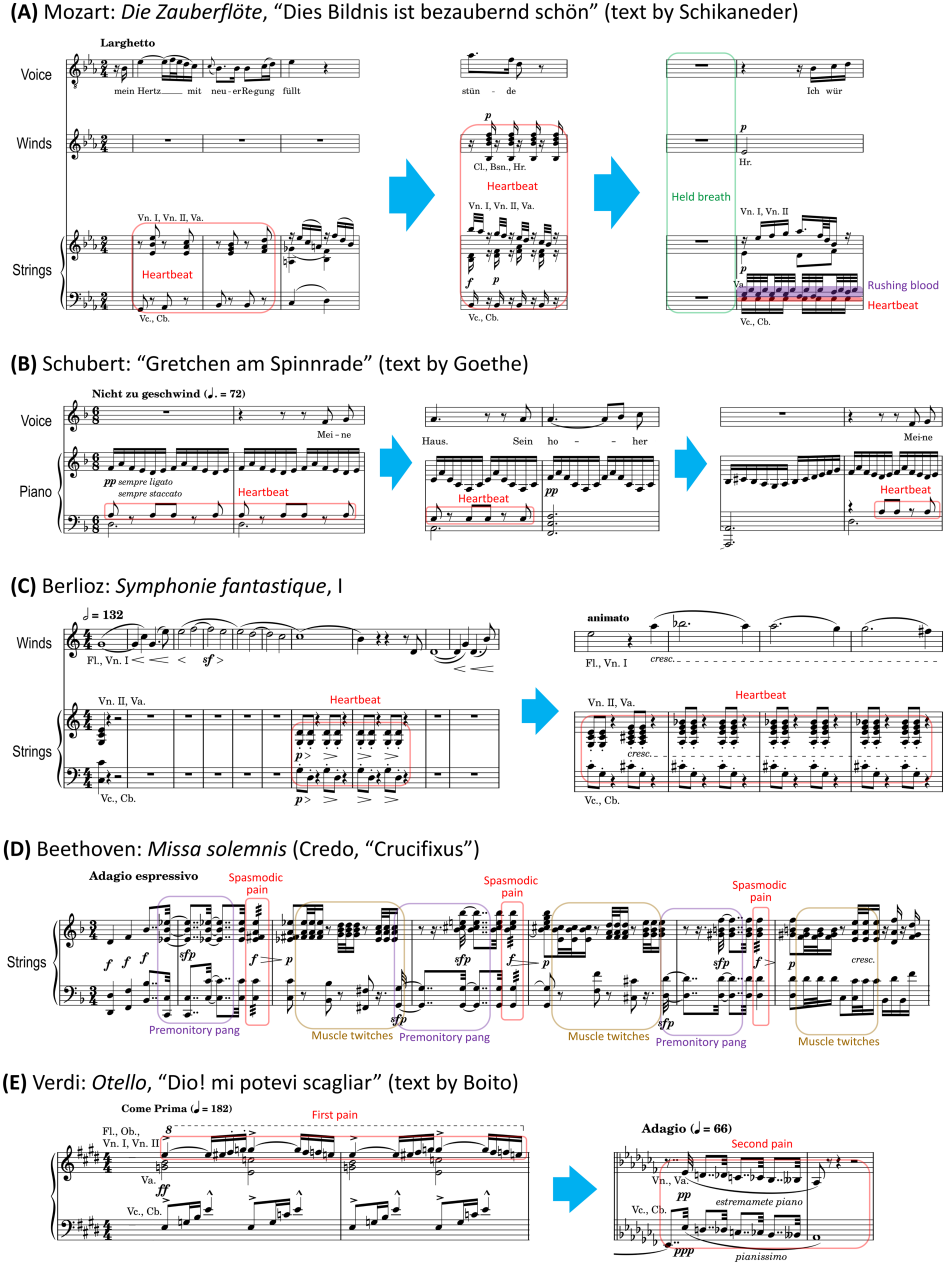
Simplified score excerpts for the selected works. Colored boxes or bars indicate pseudo-interoceptive cues; blue arrows indicate omitted measures. **(A)** Mozart, *Die Zauberflöte*, “Dies Bildnis ist bezaubernd schön.” **(B)** Schubert, “Gretchen am Spinnrade.” **(C)** Berlioz, *Symphonie fantastique*, I. **(D)** Beethoven, *Missa solemnis* (strings only). **(E)** Verdi, *Otello*, “Dio! Mi potevi scagliar” (strings and woodwinds only). Fl., flute; Ob., oboe; Cl., clarinet; Bsn., bassoon; Hr., horn; Vn., violin; Va., viola; Vc., violoncello; Cb., contrabass (sounding an octave below written pitch).

Immediately before he resolves to seek out the woman in the portrait and press her to his “hot bosom,” Mozart inserts a brief rest that may depict a held breath. In predictive coding terms, this silence functions as a prediction error, momentarily interrupting the established heartbeat-like pattern and sharpening the listener’s expectations. Following this, over the rapid, pulsating rhythm in the lower strings, the agitated viola figurations add a layer of textural turbulence that can be likened to blood rushing through the body’s vessels (Figure 2A).

### 4.2 Schubert, “Gretchen am Spinnrade”: anxious heartbeat and fantasized relief

Schubert’s “Gretchen am Spinnrade” (D. 118) is often cited as a paradigmatic example of musical onomatopoeia: the right-hand perpetual-motion figure represents the spinning wheel, while the left-hand bass suggests the treadle. From a psychosomatic perspective, however, this spinning-wheel figuration can also be heard as externalizing Gretchen’s ruminative thought patterns. In particular, the inner pulsation in the middle register seems to function as a “heartbeat layer” that contributes to the overall sense of anxious unease. This interpretation aligns with Seth and Friston’s (2016) active inference account, in which precision is implemented physiologically via neuronal gain or neuromodulation. They propose that anxiety and psychosomatic conditions involve aberrant precision weighting assigned to threat-related interoceptive signals. In this light, the relentless musical pulsation simulates Gretchen’s state of interoceptive hypersensitivity, where the subject becomes unable to attenuate the precision of cardiac signals due to the profound uncertainty and turmoil of her romantic obsession.

A crucial turning point occurs in the central major-mode episode, where Gretchen rapturously describes Faust and ultimately fantasizes about his kiss. At this point, the left-hand accompaniment thins out: the steady treadle-like motion and the heartbeat layer merge into sustained chords. For an empathic listener adopting Gretchen’s perspective, this may feel as if attention is drawn away from the chest into an episodic simulation of Faust’s presence and kiss, with the intrusive heartbeat entirely disappearing from awareness. When the music returns to the minor mode, however, the anxious heartbeat re-emerges. It seems that attention is pulled back from sweet fantasy to stagnant reality (Figure 2B).

### 4.3 Berlioz, *Symphonie fantastique*, I: pathological cardiac pulsation

Berlioz’s unrequited love for a young actress produced severe nervous overstimulation, trembling, and a painful hypersensitivity of all his faculties; in his own account, he described listening to his heartbeat, with its pulsations shaking him “like the pistons of a steam engine” (Brittan, 2006). In the first movement of his *Symphonie fantastique*, the *idée fixe* is interleaved with low-string pulsations that gradually intensify over the course of the exposition.

These pulsations, which initially intrude between thematic phrases, can be interpreted as a musical analogue of palpitations breaking into conscious thought. The lyrical, upward-striving contour of the idée fixe conveys longing and obsession. However, the increasingly agitated pulsations—evolving from intermittent intrusions into a pervasive undercurrent—can be metaphorically understood as a loss of autonomic control (Figure 2C). Remarkably, even as the music wavers between major and minor modes, mirroring the lover’s anxious vacillation between hope and despair and rendering harmonic predictions uncertain, the relentless pulsations retain high precision and continue to capture the listener’s attention.

### 4.4 Beethoven, *Missa solemnis* (credo, “Crucifixus”): paroxysmal, spasm-like pain

Beethoven’s lifelong struggles with illness and pain are well documented, and his works sometimes translate physical suffering into musical terms. In the “Crucifixus” section of the Credo in the *Missa solemnis*, he deploys harmony, dynamics, and recurring rhythmic patterns in a way that vividly evokes bodily torment (Drabkin and Societies, 1991). In the strings, the texture is punctuated by three dissonant chordal attacks. While their harmonic content grows increasingly tense and tonally ambiguous (low precision), their temporal organization remains strictly regular (high precision) (Figure 2D). This juxtaposition generates a high level of pwPE, thereby commanding the listener’s attention.

Importantly, this musical texture mirrors the complex temporal profile of somatic distress. Physiologically, the sonorities function as a musical analogue for paroxysmal, spasm-like pain. The onset of a spasmodic episode is heralded by intermittent sharp accents—akin to premonitory pangs (Thiarawat et al., 2016)—which are likely realized here through syncopated sforzando attacks in the strings. Subsequently, rapid thirty-second-note repetitions emulate the main spasmodic attack, capturing the tremulous, cramping quality characteristic of such pain (Satoyoshi and Yamada, 1967; Wallace et al., 2014). Finally, softer, fragmented figures may simulate the sensation of muscle fasciculations (involuntary twitches) or micro-convulsions that often follow spasmodic pain attacks (Dewarrat et al., 1994). This string accompaniment thus maps the theology of the Passion—articulated by the vocal lines—onto an interoceptive landscape of escalating bodily distress.

### 4.5 Verdi, *Otello* (“Dio! mi potevi scagliar”): first and second pain

In *Otello*’s Act III monologue “Dio! mi potevi scagliar,” Verdi uses visceral orchestral writing to depict the tragic hero’s torment (Busch and Verdi, 1988). From a physiological perspective, the passage offers a striking musical analogue to the biphasic phenomenology of pain. The orchestral prelude can be heard as an artistic dramatization of two experiential phases often distinguished in pain theory: an initial, sharp “first pain” mediated by A-*δ* fibers, followed by a slower, more diffuse “second pain” mediated by C fibers (Strigo and Craig, 2016). Rather than literally mirroring the rapid time course of A-δ and C-fiber signaling, the music dilates this two-stage profile onto a perceptually accessible timescale, inviting the listener to inhabit a prolonged, dramatized transition from acute, piercing pain to lingering torment.

The passage opens with high-register string attacks: *staccato fortissimo* gestures that strike with sudden force. These piercing blows can be likened to the rapid, well-localized aspect of first pain—an abrupt, shock-like intrusion that commands immediate attention. As a tonally ambiguous motif repeats with grim regularity and descends in register, these high-register jolts give way to syncopated figures in the middle and lower registers, followed by a chromatic, slowly descending line and suspended harmonies. This final phase evokes a slower, pervasive suffering reminiscent of second pain: a duller and poorly localized ache that is entwined with psychological despair (Figure 2E).

### 4.6 Summary of the illustrative analyses

These analyses illustrate how listeners can hear dynamic musical shifts as state transitions within a virtual body, thereby fostering deep immersion in the work’s emotional landscape. This immersion is likely supported by the hierarchical nature of the internal generative model, where distinct neural layers process predictions over different time scales. Converging theoretical and empirical work suggests that higher levels of the neural hierarchy integrate information and generate predictions over progressively longer time scales (Hasson et al., 2015; Friston et al., 2017b; Schmitt et al., 2021; Tsai, 2024). While the musical-affective changes discussed above elicit prediction errors that drive the updating of lower-level beliefs regarding the virtual body’s momentary somatic state, the implications at higher levels are distinct. Higher-level layers, which encode predictions over longer time spans, treat these persistent streams of lower-level prediction errors not as failures of the model, but as evidence substantiating the volatile nature of the musical persona. Thus, rather than disrupting the system, these local surprises consolidate the reality of the persona inhabiting the virtual world, deepening the listener’s engagement with the unfolding narrative.

Fundamentally, the validity of this framework does not hinge on the listener’s actual physiological state closely tracking the interoceptive drama depicted in the music. As the examples from Berlioz, Beethoven, and Verdi suggest, the virtual body sometimes undergoes pathological arousal or physical torment—states that the listener is unlikely, and arguably unwilling, to embody in full. Instead, the proposal is that listeners engage in a form of affective and motor simulation, integrating sensory evidence from the music, extramusical context, and their own schematic knowledge of emotion. In light of the Theory of Constructed Emotion (Barrett, 2017), such listening episodes can be seen as opportunities to refine and enrich the brain’s emotion concepts. By simulating extreme negative states at an abstract level, music deepens the listener’s epistemic grasp of life’s hardships and suffering, allowing profound meanings to be constructed (Menninghaus et al., 2017; Li and Tsai, 2025).

## 5 Discussion

Recent research on musical emotion has produced a substantial body of experimental data; however, musical stimuli are still frequently described in broad categorical terms (e.g., happy, sad, fast, or slow), leaving underspecified how particular musical patterns map onto specific bodily sensations. In this article, musical emotion is treated as a special case of active interoceptive inference, a perspective that affords granular mappings between discrete musical features and virtual bodily states. Musical cues act as pseudo-interoceptive evidence, and felt emotion arises as listeners’ generative models minimize prediction error with respect to a musically implied virtual body. At this stage, the account is offered as a conceptual framework: the correspondences proposed are probabilistic tendencies, intended to guide future empirical tests.

Conceptually, this framework distinguishes itself by foregrounding top-down active inference, thereby extending accounts that emphasize bottom-up embodied mechanisms—specifically, the action-program account of Habibi and Damasio (2014) and the rhythmic entrainment and emotional contagion components of Juslin’s (2013) unified theory. In these specific mechanisms, musical structure is typically taken to drive bodily changes and affect via stimulus-driven synchronization and peripheral feedback. Here, by contrast, bodily arousal is treated as a necessary but nonspecific energetic substrate: the listener’s real body constrains what states are plausible, yet the experienced quality of the emotion depends on an inferential interpretation. Specifically, the musically implied virtual body supplies a qualitative frame through which arousal is parsed into particular affective–somatic states, shifting the explanatory emphasis from “what the music makes the body do” to “what bodily state the brain infers, given musical evidence and prior expectations.” For instance, in *Symphonie fantastique*, insistent pulsations may increase real physiological arousal, yet the listener need not literally entrain to the beat. The resulting conscious experience can be understood as a composite, blending proprioceptive and motoric components (e.g., subvocal rehearsal) with ongoing visceral background, while additionally incorporating simulated cardiac rhythms attributed to the musically implied virtual body.

This difference can be situated within the hierarchical organization of interoception. Lower-level brainstem pathways continue to regulate homeostasis, while insular subregions form a graded interface between bodily physiology and higher-level affective integration. In particular, the posterior insula can be construed as a relay where sensory evidence—including acoustically structured, pseudo-interoceptive cues—can be integrated with ongoing bodily signals and made available to higher-order regions such as the anterior insula. The anterior insula, in turn, supports integrative representations that yield coherent affective–interoceptive inferences about the state of the virtual body. In this way, the framework offers a distinctive mechanism-level claim that complements prior theories: music can shape emotion by selectively weighting and reinterpreting bodily evidence within an interoceptive generative model, thereby generating a controlled, perceptual-affective “hallucination” of bodily change without necessarily perturbing the physiological integrity of the real body.

A longstanding issue in music–emotion research concerns how to characterize the affective states that music evokes, and whether they are “genuine” emotions continuous with everyday life or distinctively “aesthetic” forms of feeling. Juslin and Västfjäll (2008) argued for continuity, whereas Kivy (1990) argued that our response to purely musical structure constitutes a distinctive form of “being moved” that should not be conflated with garden-variety emotions. The present account offers a mechanistic middle ground: physiological arousal in the listener’s real body can be genuine and consequential, while its qualitative interpretation is shaped by inference, because musical structure supplies pseudo-interoceptive evidence that supports a musically implied virtual body—a bodily hypothesis that frames how arousal is experienced. Music-induced affect can therefore be both real (in energetic and autonomic terms) and virtual (in the inferred bodily hypothesis that organizes subjective feeling), clarifying how *as-if* experience can arise from ordinary interoceptive mechanisms under sensory constraint. This perspective also reframes how listeners can enjoy negative emotions in music (Levinson, 1982) through aesthetic distancing (Menninghaus et al., 2017). The virtual body provides a mechanistic instantiation of “distance,” enabling salient affective–interoceptive qualia without obligatorily recruiting the full set of real-world action policies or corresponding physiological perturbations. In this way, long-standing aesthetic questions could be recast in active-inference terms of policy selection, precision control, and bodily hypotheses.

Beyond explaining how music evokes affect-laden bodily simulations, this framework also extends previous predictive-coding accounts of groove. Vuust et al. (2018) argued that, in the context of syncopation, bodily movement serves to reinforce the internal pulse and resolve rhythmic uncertainty. By contrast, this article foregrounds affective mimicry as a mechanism for minimizing interoceptive prediction error, shifting the analytic focus to the semantic significance of specific pseudo-interoceptive cues. This principle of simulating specific bodily states aligns closely with the predictive coding model of groove proposed by Tsai (in press). Focusing on low-frequency, low-complexity rhythmic patterns, this model argues that the kick drum and bass approximate the multisensory consequences of locomotion—providing footfall-like impact vibrations, vestibular fluctuations, and weight-shifting patterns. When the listener remains physically still, a mismatch arises between this auditory simulation of locomotion and sensory evidence that the body is at rest. The system reduces this prediction error by engaging in covert motor simulation—*as-if* actions experienced subjectively as the sensation of groove. Crucially, both the present framework and my groove model (Tsai, in press) posit that active inference is anchored in specific internal bodily sensations. Furthermore, both highlight the critical role of timbre—particularly low-frequency energy—in conveying somatic meaning. When a rhythmic pattern delivered via such timbre appears in a musical work, the simulation of locomotion and affective mimicry likely occur simultaneously, fusing into a composite experience of an animated, feeling virtual body.

Building on “mimetic” (Molnar-Szakacs and Overy, 2006; Cox, 2016) and “emotion contagion” (Juslin, 2013) accounts of music, this article advances the field in two key respects. First, it broadens the scope of internal simulation beyond the commonly discussed domain of laryngeal proprioception—specifically, the imagined vocal-fold tension required to produce pitch—to include cardiovascular and pain-like sensations. This perspective underscores the critical role of the accompaniment. While high-register principal melodies typically command attention and invite subvocal mimicry, the underlying, relatively unobtrusive accompaniment covertly shapes the emotional landscape, particularly by simulating the cardiac and motor rhythms of the musical persona.

Second, this article offers a computational rationale for embodied simulation accounts of music: we do not mimic simply for the sake of imitation, but because covert simulation enables the brain to interpret sensory signals more efficiently. From an evolutionary perspective, the neural mechanisms underlying such internal mimicry can be situated within the framework of *exaptation*—the reuse or “repurposing” of traits that originally evolved for one function but were later co-opted for another (Gould and Vrba, 1982). The sensorimotor system serves as a canonical example: in primates, premotor and parietal circuits likely evolved to support fine-grained sensorimotor control and were later “reused” for action understanding and imitation (Kilner et al., 2007; Hurley, 2008). A similar exaptive logic can be applied to the interoceptive network, which operates as a control system for the internal milieu and has arguably been repurposed from basic homeostatic regulation to support empathy and theory of mind (Ondobaka et al., 2017). I propose that musical experience constitutes a further reuse of this architecture: music recruits this same circuitry anew to track the state of a virtual body implied by sound.

Although the case studies in this article focus on works with text or programmatic descriptions, the proposed mechanism of active interoceptive inference is likely not limited to such contexts. Even in the absence of explicit extramusical information, listeners may still recruit generative models to interpret “absolute” music. For instance, listeners familiar with the classical style can readily associate eighteenth-century “sigh” figures with specific respiratory patterns, using this schematic knowledge to infer specific affective states. Similarly, the *Sturm und Drang* style—characterized by rapid, low-frequency pulsations in the minor mode, as seen in the opening themes of Mozart’s Symphony No. 25 and Haydn’s Symphony No. 45—can directly simulate the cardio-respiratory signatures of anxiety or fear. Indeed, Spitzer’s (2010) analysis of fearful feelings in Schubert’s *Unfinished Symphony* demonstrates how pseudo-interoceptive cues can drive affective interpretation purely through musical structure, without reliance on extramusical framing. Furthermore, research indicates that instrumental music frequently evokes visual imagery (Juslin, 2013; Presicce and Bailes, 2019), which likely entails an embodied, interoceptive dimension. Thus, it is plausible to posit that active interoceptive inference remains a primary engine of emotion even in purely instrumental contexts.

A key boundary condition concerns the cultural specificity of the proposed music–interoceptive mappings. Some dimensions of musical structure are likely to be shaped to a greater extent by learned conventions and stylistic enculturation. Harmonic syntax and major–minor tonality are a paradigmatic case: their affective connotations plausibly depend on historically contingent compositional norms and culturally transmitted listening schemata. By contrast, other dimensions—most notably rhythm, tempo, periodicity, and intensity dynamics—may be comparatively more constrained by general perceptual and sensorimotor priors, and thus more likely to support cross-cultural mappings to arousal and action-readiness. A useful illustration comes from Chinese *xiqu*, where a practice known as *jinla manchang* (also *jinda manchang*; “tight accompaniment, slow/free singing”) is widely used—particularly in Yue opera—to depict heightened agitation or emotional escalation while preserving a stretched, quasi-recitative vocal delivery. In this texture, a fast, regular instrumental/percussive pulse (often organized at one or two beats per bar) coexists with a freer, elongated vocal line, yielding two concurrent temporal streams. Within the present framework, the “tight” accompaniment can be construed as pseudo-interoceptive evidence with cardiac-like signatures, whereas the freer vocal layer sustains higher-level narrative and evaluative structure that constrains how arousal is interpreted. This contrast underscores an empirical agenda: mappings grounded in tonal-harmonic conventions should show stronger dependence on cultural familiarity, whereas mappings grounded in rhythmic/tempo cues should generalize more broadly, primarily modulated by context and attentional set.

For musical cues to function as pseudo-interoceptive evidence, they must engage neural circuitry that links auditory representations to interoceptive and affective processing—most notably, pathways between the auditory cortex and the insula. This structural and functional coupling is well documented in neuroimaging research. Diffusion-weighted imaging shows that the structural integrity of white-matter tracts connecting auditory cortex and insula predicts individual differences in musical reward and aesthetic sensitivity (Sachs et al., 2016). Converging fMRI findings likewise suggest that these pathways are crucial for turning acoustic structure into embodied, affect-laden experience: during listening to joyful versus fearful music, emotion-specific functional connectivity emerges between primary and secondary auditory cortices and granular regions of the insula (Koelsch et al., 2021). The insula’s role as a hub of the salience network (Menon and Uddin, 2010) provides an additional layer of explanation for why certain musical events feel particularly gripping. During music listening, functional coupling between auditory cortex and the insula is thought to support the selection of acoustically and emotionally salient events (Putkinen et al., 2021).

Long-term musical experience appears to enhance the efficiency of “sound–body–emotion” integration. Professional musicians show increased anterior insula connectivity with networks supporting empathy, attention, and sensorimotor integration (Zamorano et al., 2017), and this connectivity correlates with higher empathic ability and affective sensitivity (Gujing et al., 2019). Such plasticity is not limited to professional musicians. Older adults with musical experience show stronger dorsal anterior insula–sensorimotor coupling, consistent with reinforced somatic awareness (Ai et al., 2022), and singing training selectively enhances bilateral anterior insula connectivity with speech–sensorimotor networks (Zamorano et al., 2023). Moreover, He et al. (2017) found that a longitudinal music intervention in patients with schizophrenia increased functional connectivity between anterior insula and anterior cingulate cortex, as well as between posterior insula and sensorimotor cortices. Together, these findings suggest that music can shape, and in some cases partially repair, the neural circuitry that integrates internal bodily states with external sensory cues.

Music therapy provides both a testing ground for, and an application of, the proposed mappings between specific musical parameters and interoceptive sensations. For instance, the Therapeutic Function of Music framework systematically links acoustic features such as tempo, contour, and dynamics to targeted levels of physiological arousal and emotion regulation, demonstrating that carefully structured musical elements can modulate bodily state via bottom-up mechanisms (Sena Moore and Hanson-Abromeit, 2015). Extending this logic, adult music listening can be understood as a form of self-administered interoceptive training, in which listeners repeatedly experience musical trajectories from tension or crisis toward resolution and recovery. Specifically, this engagement may enhance interoceptive sensibility—the subjective tendency to focus on and appraise somatic states—and sharpen interoceptive awareness by continuously recalibrating the generative models that underpin our conscious feeling of the body. Significantly, in contemporary media environments, problematic smartphone use and addiction-like social media engagement have been associated with reduced insula gray-matter volume and altered salience-network connectivity (Turel et al., 2018; Lee et al., 2024). In this context, sustained engagement with musical narratives—where interoceptive states unfold over extended timeframes—offers a vital alternative mode of experience. By continuously exercising and recalibrating interoceptive predictive models to resolve high pwPEs, such listening habits may potentially counteract these deficits and strengthen capacities for interoceptive awareness and regulation.

The proposed mappings between specific musical parameters and interoceptive sensations also invite a rethinking of musical listening, performance, and interpretation. If musical emotion is partly grounded in listeners’ schematic knowledge of interoceptive states, affective responses to music need not be regarded as purely intuitive or fixed. Listeners and performers can, in principle, learn to associate particular musical gestures with particular interoceptive qualities. Such learning is likely facilitated by basic music-analytic and physiological knowledge, together with enriched extramusical information. On this view, sensitivity to the interoceptive dimensions of music is a trainable skill. Future studies could test this claim by comparing behavioral reports, peripheral physiological responses, and neural markers of interoceptive–auditory processing before and after targeted training in recognizing specific musical cues.

Complementary experiments could systematically manipulate cardiac-like versus pain-like cues within tightly controlled musical stimuli to determine whether—and through which pathways—these features modulate canonical markers of interoceptive inference. A recent intracranial electrophysiology study (Banks et al., 2023) derived a low-dimensional “functional geometry” of auditory cortical resting-state networks and showed that the posterior insula occupies an intermediate position between auditory cortex and limbic structures in the resulting embedding. Banks et al. (2023) further proposed that, given its robust auditory responsiveness, the posterior insula may help transform auditory cortical information into affective representations in the anterior insula, consistent with a linking role between auditory and limbic systems. While resting-state geometry cannot establish directionality, it helps sharpen a testable hypothesis: pseudo-interoceptive musical cues should reconfigure effective connectivity. Specifically, they should enhance coupling between auditory cortex and posterior insula, and strengthen posterior-insula interactions with the anterior insula and anterior cingulate cortex. This pattern would be consistent with acoustic evidence being converted into affectively salient interoceptive representations and subjective qualia. These predictions can be tested by combining connectivity models with concurrent autonomic measures. Cardiac-like cues should preferentially bias cardiovascular-related bodily hypotheses and autonomic readiness, whereas pain-like cues should more strongly engage insula–cingulate pathways linked to salience, aversive qualia, and protective bodily predictions.

The network-level organization reported in Banks et al. (2023) also offers a principled way to distinguish what is driven by musical structure from what is driven by contextual priors. Their findings place auditory cortex in close functional proximity to a limbic–semantic constellation that includes the temporal pole and medial temporal lobe structures, providing an anatomically plausible route through which semantic and mnemonic information can shape auditory inference. This aligns with task-based evidence linking the temporal pole to context-sensitive socioemotional integration (Pehrs et al., 2014; Pehrs et al., 2018; Li et al., 2019). Accordingly, an empirically tractable approach is to hold the acoustic stimulus constant while manipulating biographical knowledge, textual meaning, or programmatic narratives as contextual primes that vary the precision of higher-level priors. Mechanistically, precision weighting can be framed more simply as context-dependent gain modulation of prediction-error signaling and belief updating, which should be observable as systematic changes in directed coupling between auditory cortex, posterior insula, temporal pole/medial temporal circuitry, and anterior insula/anterior cingulate cortex. On this account, contextual primes should produce shifts in (i) the relative coupling between auditory cortex and posterior insula versus coupling between temporal pole/medial temporal circuitry and anterior insula/anterior cingulate cortex, (ii) the relative weighting of cardiac-like versus pain-like bodily hypotheses inferred from the same musical input, and (iii) the correspondence between subjective interoceptive qualia and peripheral physiology.

## 6 Conclusion

This article proposes an active interoceptive inference framework wherein musical cues function as pseudo-interoceptive evidence, prompting a “controlled hallucination” of bodily change. On this view, listeners recruit generative models to infer the state of a virtual body, with felt emotion arising as these models minimize prediction error. Admittedly, this framework captures only part of the multifaceted ways in which music moves us; mechanisms such as memory, reward, and social meaning undoubtedly interact with interoceptive inference in ways that warrant further investigation. Moreover, while the musical analyses presented here focus on Western art music and on cardiac- and pain-like qualia, future work should explore whether similar principles extend to other genres, cultures, and somatic domains—including respiratory rhythms, thermal sensations, muscular tension, vibrotactile roughness (e.g., the tingling, buzzing sensation often reported with rock music), and even experiences of weightlessness.

## Data Availability

The original contributions presented in the study are included in the article, further inquiries can be directed to the corresponding author/s.
